# Molecular Engineering Mediated Interfacial Assembly as an Artificial Extracellular Matrix Remolds Bacteria With Enhanced Abiotic Resilience

**DOI:** 10.1002/advs.75937

**Published:** 2026-06-03

**Authors:** Yuanyuan Wang, Zili Jia, Yang Li, Qitao Wang, Shuai Hou, Lei Liu

**Affiliations:** ^1^ Institute for Advanced Materials School of Materials Science and Engineering Jiangsu University Zhenjiang China

**Keywords:** artificial extracellular matrix, biomimetic nanocoating, desiccation tolerance, interfacial assembly, microbial biocontrol

## Abstract

Microbial inoculants are central to sustainable agriculture; however, the vulnerability of bacterial cells to desiccation represents a fundamental barrier to their effective use in open‐environment applications. While nature employs extracellular polymeric substances for protection, synthetic replication of this multifunctional, nanoscale interface remains a challenge. Here, we report a biomimetic strategy to assemble an artificial extracellular matrix (AEM) directly on the surface of *Pseudomonas fluorescens*, conferring exceptional abiotic resilience. Inspired by amyloid‐protein architecture in natural biofilms, we engineered an interfacial coating via the conformational transition of lysozyme into a β‐sheet‐rich, adhesive scaffold, which electrostatically co‐assembles with alginate polysaccharides at the cell envelope. This conformal nanocoating provides dual‐mode protection: it acts as a viscoelastic hydration buffer that prevents membrane rupture, and it elicits a transcriptional response that upregulates genes associated with respiration, osmoprotection, and proteostasis. Optimized at a 1:1 protein‐to‐polysaccharide ratio, the AEM enhances bacterial survival after desiccation by 30.9‐fold. Furthermore, it enables robust seed adhesion and storage stability, translating into effective biocontrol against *Fusarium* pathogens in a model agricultural system. This work establishes a versatile strategy for programming cellular interfaces, bridging materials design and microbial functionality to engineer resilient living systems for real‐world deployment.

## Graphical abstract

A biomimetic artificial extracellular matrix is assembled directly on *Pseudomonas fluorescens* by co‐assembling amyloid‐like lysozyme and alginate at the cell surface. This conformal nanocoating acts as both a hydration buffer and a physiological priming layer, markedly enhancing desiccation tolerance, seed adhesion, storage stability, and biocontrol efficacy against *Fusarium* in agriculture.

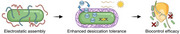

## 1 Introduction

With the escalating demand for sustainable agriculture, microbial inoculants have emerged as a core pillar of modern agroecosystems [1, 2, 3]. Despite their potential to replace synthetic chemicals, the transition from controlled laboratory settings to field applications is frequently hindered by poor operational consistency and environmental instability. Among these challenges, desiccation stress represents a critical bottleneck; the rapid loss of water during product formulation, storage, and environmental exposure typically leads to a catastrophic decline in bacterial viability. For living matter, water loss constitutes a profound thermodynamic crisis: dehydration collapses macromolecular structure, disrupts membranes and metabolism, and induces oxidative stress [4, 5]. This limitation is not merely a technical obstacle to long‐term preservation but a fundamental barrier to the deployment of viable microbial technologies in fluctuating open environments. While genetic engineering can be used to rewire internal stress‐response pathways [6], such approaches are organism‐specific, metabolically burdensome, and face significant regulatory hurdles for environmental release. Current non‐genetic strategies to enhance desiccation tolerance rely predominantly on macroscopic encapsulation (e.g., hydrogel beads) or bulk formulation methods such as freeze–drying with cryoprotectants [7, 8, 9]. While these synthetic scaffolds provide macroscale support, they lack the structural precision required to establish the nanoscale interfaces necessary to withstand complex physical and chemical perturbations at the cellular level.

To enhance the abiotic resilience of beneficial microbes, substantial efforts have been directed toward engineering nanoscale protective surface coatings [10, 11], including polymers [12, 13], lipid membranes [14], metal‐phenolic networks [15, 16, 17], metal‐organic frameworks [18], and nanozymes [19]. For example, metal‐phenolic networks and related supramolecular assemblies effectively protect bacteria from antibiotics, oxidative stress, and thermal fluctuations by forming dense, conformal shells that physically isolate cells from hostile surroundings [15, 16, 17]. Similarly, mineralized coatings composed of calcium salts provide resistance to acidic environment and organic solvent by mechanically stabilizing the cell envelope and restricting mass transport [20, 21]. Despite these advances, most existing approaches rely on rigid barriers that primarily function through passive shielding. Effectively mitigating desiccation stress necessitates a more sophisticated consideration of material properties, particularly the transition from rigid protection to soft, hydrated interfaces [22]. Inspiration can be drawn from xerotolerant bacteria and biofilms, which achieve exceptional desiccation tolerance by secreting complex extracellular polymeric substances (EPS) [5, 23, 24, 25]. Rather than a single‐component barrier, EPS is a sophisticated composite matrix of polysaccharides, proteins, and nucleic acids [26]. Within this architecture, polysaccharides function as hydrated, viscoelastic scaffolds that retain water and buffer mechanical stress, while proteinaceous components, often adopting amyloid‐like conformations, serve as structural crosslinkers that reinforce cohesion strength and mediate interfacial adhesion [27, 28, 29]. Together, they form a soft, hydrated interface capable of dissipating mechanical strain, retarding water efflux, and coordinating cellular responses. Replicating such multicomponent, multifunctional architectures at the immediate cell–environment interface remains a fundamental challenge.

**FIGURE 1:**
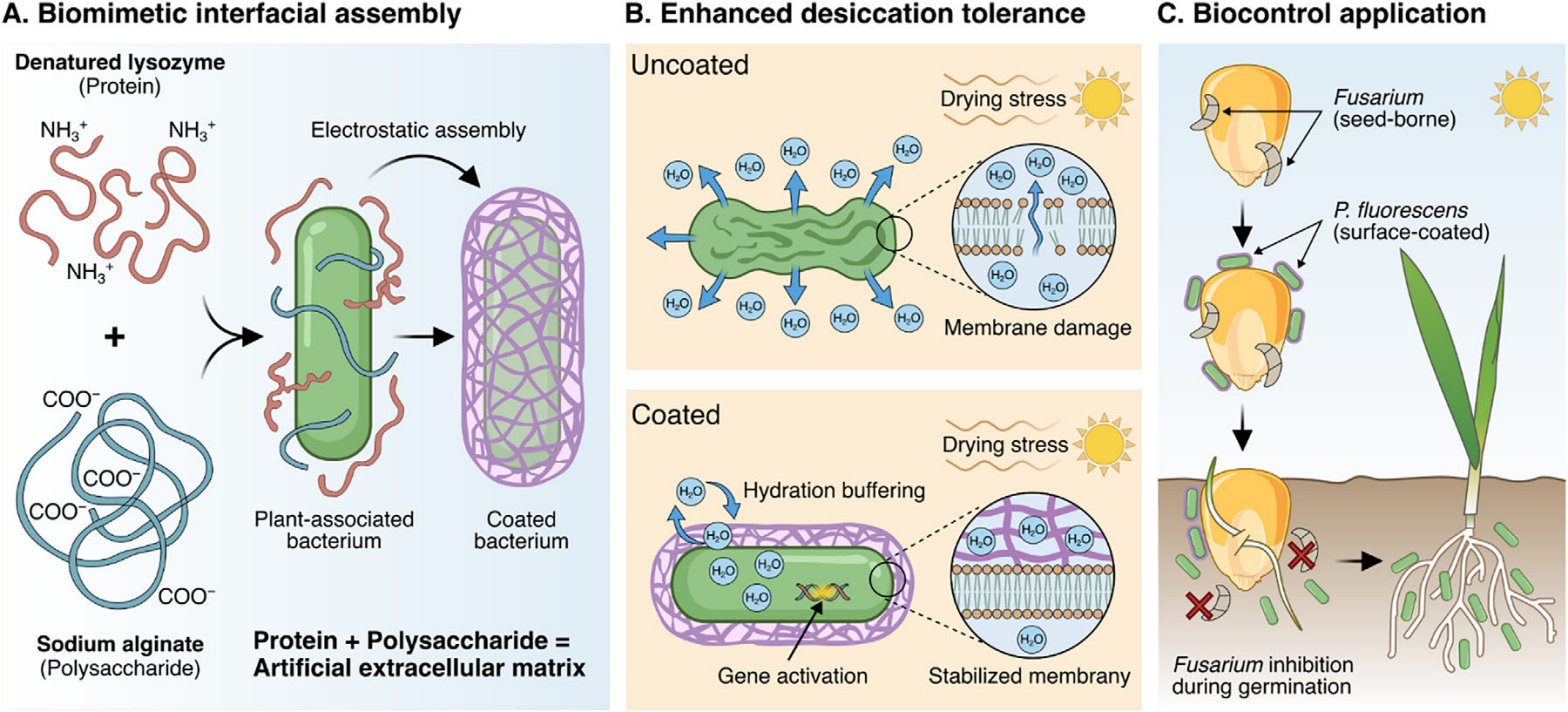
Schematic illustration of the biomimetic interfacial assembly and its functional consequences. (A) Interfacial assembly strategy: Denatured lysozyme (positively charged) and sodium alginate (negatively charged) co‐assemble on the surface of plant‐associated bacteria (*Pseudomonas fluorescens*) via electrostatic attraction to form an artificial extracellular matrix. (B) Mechanism of desiccation tolerance: Under drying stress, uncoated bacteria suffer from rapid water efflux and membrane damage. In contrast, the coated bacteria benefit from hydration buffering provided by the matrix, which stabilizes the membrane and triggers gene activation for stress resistance. (C) Biocontrol application: The surface coating enhances bacterial adhesion to corn seeds. The coated bacteria survive desiccation and effectively inhibit *Fusarium* infection during seed germination.

Herein, we report a biomimetic strategy to assemble an artificial extracellular matrix (AEM) directly on the surface of plant‐associated bacteria (*Pseudomonas fluorescens*), endowing them with exceptional desiccation tolerance (Figure 1). Guided by atomistic molecular dynamics (MD) simulations, we developed an amyloid‐like adhesive protein scaffold that electrostatically co‐assembles with alginate polysaccharides at the bacterial surface to create a conformal 20 nm thick nanocoating. This AEM provides a dual‐mode protection: it acts as a physical hydration buffer to prevent membrane rupture and simultaneously triggers a coordinated transcriptional response, ultimately enhancing bacterial survival by over 30.9‐fold under acute desiccation. Crucially, as a microbial inoculant applied to corn seeds, the AEM ensured robust seed adhesion and enabled 3‐month storage stability under desiccation, facilitating effective biocontrol against seed‐borne *Fusarium* pathogens in a model agriculture system. By directly linking molecular design with microbial physiology, this work establishes a versatile and generalizable route to engineer functional cellular interfaces for real‐world deployment.

## 2 Results and Discussion

### 2.1 Design Principle: Biomimetic Assembly of an Amyloid‐Polysaccharide Composite Matrix

Our AEM design mimics the synergistic composition of natural EPS, which combines amyloid‐like proteins with polysaccharides to form a protective, hydrated interface. We selected hen egg‐white lysozyme as the protein component and sodium alginate as the polysaccharide. Inspired by the amyloid‐like conformation of proteins in EPS, we hypothesized that converting globular lysozyme into an amyloid‐like state would enhance its adhesive properties. As supported by previous studies, breaking the native disulfide bonds of lysozyme induces a conformational transition from a compact, α‐helix‐rich fold to an extended, β‐sheet‐rich state that exhibits amyloid‐like characteristics [30, 31, 32, 33, 34]. We proposed that this denatured lysozyme, with increased backbone flexibility and exposed cationic residues, would serve as an electrostatic scaffold for recruiting anionic alginate chains to the negatively charged bacterial surface.

**FIGURE 2:**
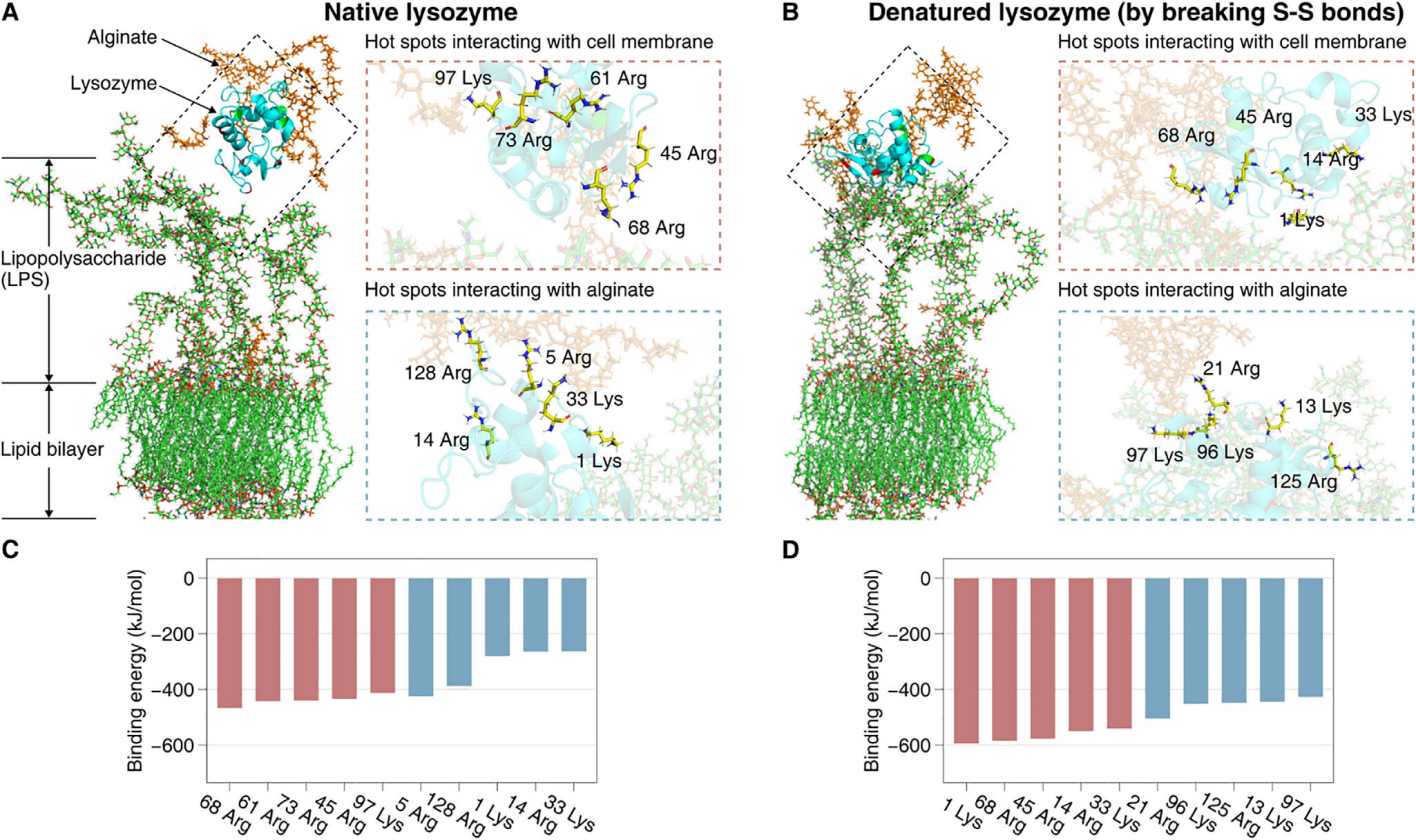
Molecular design principle and simulation of interfacial assembly. (A, B) Structural comparison from MD simulations of native lysozyme (A) and denatured lysozyme (B) interacting with the bacterial LPS layer and alginate chains. The denatured lysozyme conformation facilitates dynamic reorganization of cationic residues (Arg, Lys, shown in insets) for optimal electrostatic matching. (C, D) Calculated binding energies of the top interacting residues for native lysozyme (C) and denatured lysozyme (D), demonstrating the enhanced interfacial affinity of the denatured, amyloid‐like protein.

To validate this design rationale at the molecular level and prove the superiority of the denatured protein, we conducted atomistic MD simulations comparing native and denatured lysozyme in complex with alginate and a model bacterial lipopolysaccharide (LPS) layer (Figure 2A,B). The LPS was selected as it constitutes the primary anionic, outer‐leaflet component of the outer membrane in Gram‐negative bacteria like *P. fluorescens*, providing a physiologically relevant model of the cell surface [35]. The simulations corroborate that the conformational change is critical for enhancing interfacial assembly. While the cationic residues of native lysozyme are surface‐exposed, its rigid, globular structure restricts their optimal spatial arrangement and dynamic reorientation. In contrast, denatured lysozyme adopts a flexible, β‐sheet‐rich conformation that allows its cationic “hot spots” (e.g., Arg and Lys) to dynamically reconfigure, maximizing electrostatic complementarity with both the phosphate groups on the LPS and the carboxylate groups on the alginate chains.

Quantitative binding energy analysis from the simulations provides decisive evidence for our design choice. Denatured lysozyme exhibits a significantly stronger binding affinity to the bacterial membrane components compared to native lysozyme (Figure 2C,D and Figure S1). The interaction energy for the top five amino acid residues interacting with the LPS layer was between −500 and −600 kJ mol^−1^ for denatured lysozyme, surpassing the range for the native counterpart (−400–−450 kJ mol^−1^) by approximately 30%. Similarly, the interaction between the protein and alginate was stronger: the top five residue interaction energies were −400–−500 kJ mol^−1^ for denatured lysozyme, compared to −250–−425 kJ mol^−1^ for native lysozyme. This enhanced affinity is driven by entropic gains from increased conformational freedom and the formation of optimized, multivalent electrostatic bridges.

### 2.2 Interfacial Assembly and Characterization of the Artificial Extracellular Matrix

Guided by MD simulations, we experimentally constructed the AEM on *P. fluorescens* by incubating bacteria with a mixture of glutathione (GSH)‐denatured lysozyme and sodium alginate. A critical macroscopic observation confirmed the surface‐specific nature of the assembly. When lysozyme and alginate were mixed in solution in the absence of bacteria, rapid formation of bulk precipitates occurred due to uncontrolled electrostatic complexation (Figure S2A). In stark contrast, in the presence of bacteria, the solution remained homogeneous, with no visible precipitates (Figure S2B). This indicates that the bacterial cell envelope acts as a preferential nucleation site, effectively scavenging the oppositely charged biopolymers from solution and templating their assembly directly at the interface, thereby preventing bulk phase separation. The resulting AEM is composed of biodegradable protein–polysaccharide components that are expected to undergo natural enzymatic turnover in soil, functioning as a transient protective interface rather than a persistent coating. Although lysozyme is a known allergen, its established use in food and agricultural systems suggests that safe deployment could be achieved through standard labeling practices. Importantly, the assembly process is entirely aqueous and readily compatible with existing seed‐coating technologies.

**FIGURE 3:**
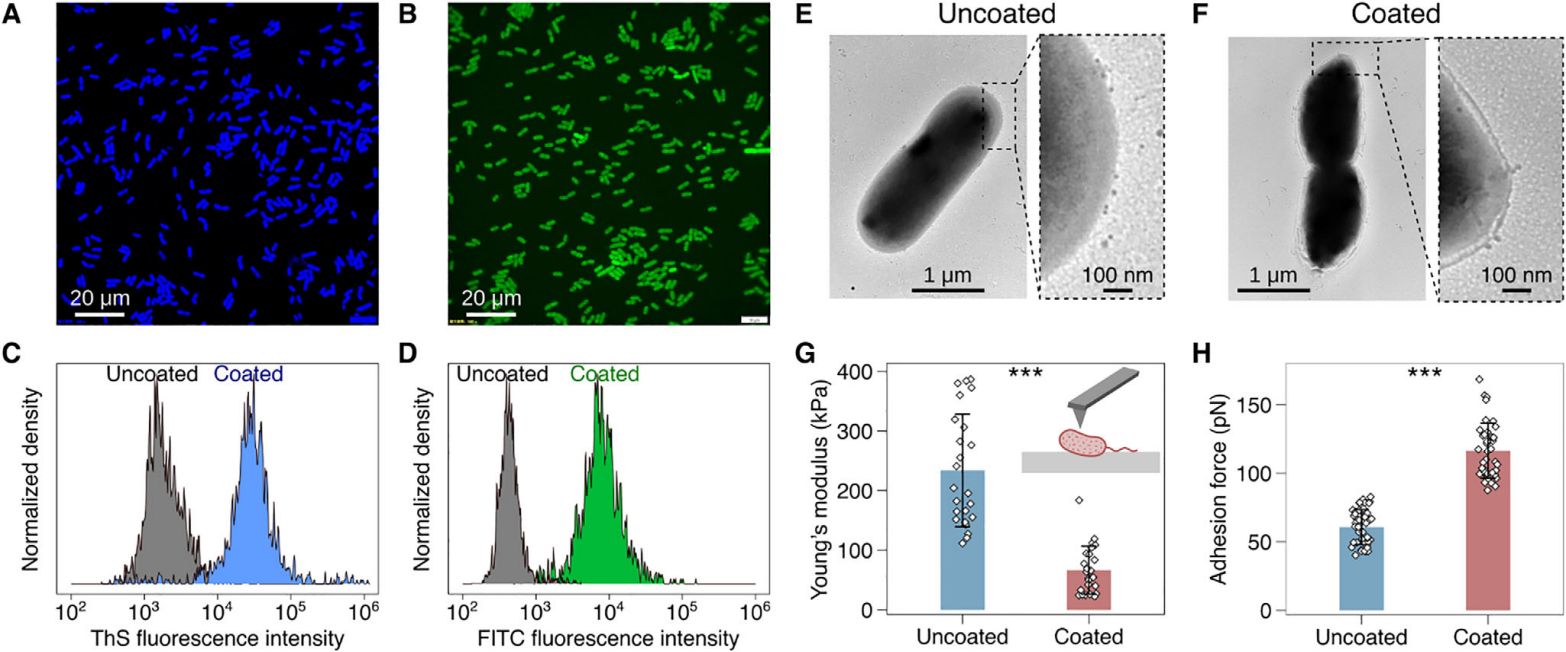
Characterization of the artificial extracellular matrix (AEM). (A, B) Fluorescence images showing AEM components: (A) Denatured lysozyme stained with Thioflavin S (blue) and (B) Alginate labeled with FITC (green). (C, D) Flow cytometry histograms quantifying the fluorescence intensity increase for coated cells (blue/green peaks) vs. uncoated controls (gray peaks). (E, F) Transmission electron microscopy (TEM) images of uncoated (E) and coated (F) bacteria. Insets show the conformal ∼20 nm thick nanofilm. (G, H) Atomic force microscopy (AFM) analysis: (G) Reduction in Young's modulus for coated cells (*n* = 24). (H) Increase in adhesion force for coated cells (*n* = 40). An unpaired two‐tailed Student's t‐test was used to compare the means of two groups. Data are presented as mean ± SD. *p < 0.05, **p < 0.01, ***p < 0.001.

To visualize and confirm the precise localization of the AEM components, we employed fluorescence microscopy. For the characterization of the protein scaffold, denatured lysozyme was stained with thioflavin S (ThS). Unlike thioflavin T (ThT), which is known to cross‐react with nucleic acids and G‐quadruplexes, ThS exhibits high binding specificity for β‐sheet‐rich, amyloid‐like structures, ensuring the signal is reflective of the proteinaceous corona rather than internal cellular components [36]. Fluorescence images revealed distinct blue fluorescence from the bacterial cells, confirming the successful surface localization of the denatured, amyloid‐like lysozyme (Figure 3A). Negligible fluorescence was observed for uncoated cells under the same staining conditions (Figure S3). In a parallel experiment, fluorescein isothiocyanate (FITC)‐labeled alginate yielded a strong, uniform green fluorescence signal on coated cells, demonstrating the co‐recruitment and integration of the polysaccharide component onto the cell envelope (Figure 3B). Control experiments with uncoated bacteria incubated with FITC‐alginate showed minimal adhesion, underscoring that the deposition is specifically driven by the intermediary of denatured lysozyme (Figure S3). The fluorescence intensity was quantified using flow cytometry. Compared to uncoated controls, coated cells exhibited a 20‐fold increase in ThS fluorescence intensity and a remarkable 400‐fold increase in FITC‐alginate signal (Figure 3C,D). This dramatic enhancement confirms that the electrostatic driving force efficiently creates a dense, surface‐confined composite layer, consistent with the multivalent binding predicted by our simulations.

The nanoscale architecture of the coating was resolved using transmission electron microscopy (TEM). While uncoated bacteria displayed clean, well‐defined cell envelopes (Figure 3E), coated cells were uniformly encompassed by a continuous nanocoating (Figure 3F). High‐magnification images revealed this film to be conformal and intimate, with a consistent thickness of approximately 20 nm. This structurally defined, yet nanoscopically thin, barrier is ideally suited to provide protection without significantly impeding molecular diffusion.

Beyond providing mere coverage, the AEM fundamentally altered the biophysical properties of the cell surface. Atomic force microscopy (AFM)‐based nanoindentation measurements revealed that the coating acts as a viscoelastic buffer. It induced a pronounced softening of the bacterial interface, reducing the Young's modulus by 72% from ∼233.8 kPa (uncoated) to ∼66.6 kPa (coated) (Figure 3G). Concurrently, the surface adhesion force nearly doubled, from 61.1 to 116.4 pN (Figure 3H). This transformation from a stiff, non‐adhesive surface to a soft, sticky interface mirrors the key mechanical attributes of natural hydrated EPS. The softness likely aids in dissipating mechanical stresses during desiccation, while the enhanced adhesiveness is crucial for substrate attachment and water retention.

### 2.3 Optimization and Enhanced Desiccation Tolerance

**FIGURE 4:**
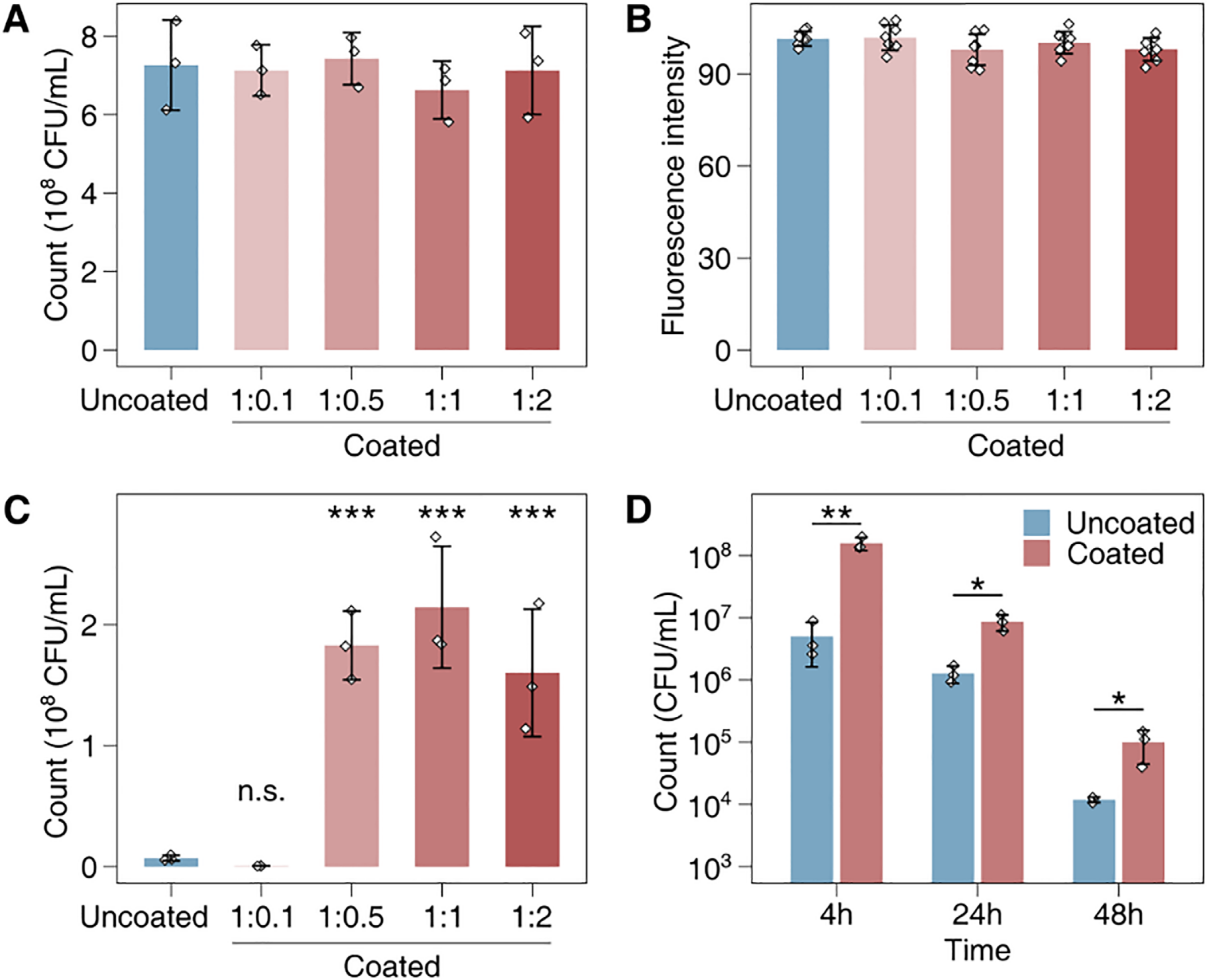
Optimization and enhanced desiccation tolerance. (A) Bacterial viability immediately after coating with denatured lysozyme/alginate complexes at different mass ratios (*n* = 3). No significant differences were observed among groups (one‐way ANOVA, *p* = 0.85). (B) Metabolic activity assessed by resazurin reduction fluorescence across coating ratios (*n* = 8). No significant differences were observed among groups (one‐way ANOVA, *p* = 0.15). (C) Survival of bacteria after desiccation stress, showing maximal recovery at the 1:1 (lysozyme:alginate) mass ratio (*n* = 3). Statistical comparisons were performed by one‐way ANOVA with Dunnett's post hoc test vs. the uncoated group. (D) Time‐resolved desiccation tolerance assay (*n* = 3). Coated bacteria (red bars) maintain high viability over 48 h, whereas uncoated bacteria (blue bars) exhibit rapid population decline. An unpaired two‐tailed Student's t‐test was used to compare coated and uncoated groups at each time point. Data are presented as mean ± SD. *p < 0.05, **p < 0.01, ***p < 0.001.

Surface engineering of living materials must be achieved without perturbing the cell's viability. We confirmed that the interfacial assembly process meets this criterion. The viability of *P. fluorescens* remained unchanged after coating with the denatured lysozyme/alginate complex, regardless of the lysozyme:alginate mass ratios tested (Figure 4A). Furthermore, resazurin reduction assays indicated that the metabolic baseline of the coated bacteria was preserved (Figure 4B), demonstrating that the AEM does not induce acute metabolic suffocation or nutrient blockade. This established a biocompatible operational window within which the composition of the AEM could be chemically optimized for maximal stress protection.

The protective efficacy of the AEM against desiccation was found to be critically dependent on the stoichiometric ratio between its protein and polysaccharide components. By systematically varying the mass ratio of lysozyme to alginate from 1:0.1 to 1:2, we identified a sharp optimum for desiccation tolerance (Figure 4C). Coatings with excess protein (1:0.1 ratio) provided minimal protection, yielding only 6.3 × 10^6^ CFU mL^−1^ post‐drying, likely due to an insufficient capacity for hydration. Increasing the alginate fraction dramatically enhanced survival, with a peak recovery of 2.1 × 10^8^ CFU mL^−1^ observed at the balanced 1:1 mass ratio. This stoichiometric dependence underscores the complementary, modular roles of the two constituents: while the amyloid‐like lysozyme acts as the indispensable electrostatic “glue” for surface anchorage, alginate serves as the essential “hydration reservoir”. The 1:1 composition appears to represent the optimal trade‐off, providing robust adhesion alongside maximal water‐retention capacity.

The kinetic resilience conferred by this optimized interface under sustained desiccation stress was exceptional. While uncoated control populations underwent catastrophic collapse within hours of drying, coated cells maintained high viability over extended periods (Figure 4D). Quantitatively, the AEM provided a 30.9‐fold survival advantage after 4 h of acute desiccation at 35°C and 35% relative humidity. Even at a more elevated temperature of 40°C, the coated bacteria still exhibited a higher survival rate than the uncoated ones by a factor of 24.3 (Figure S4). This protective effect was remarkably persistent; even after 48 h of continuous drying stress, the coated bacterial population density remained 8‐fold higher than that of uncoated cells. This sustained resilience confirms that the AEM functions not as a transient shield, but as a robust biophysical buffer that fundamentally modulates the kinetics of cellular dehydration, maintaining a protective microenvironment at the cell interface over biologically relevant timescales. Preliminary experiments with *Bacillus subtilis* further confirmed the generality of the AEM strategy, showing successful coating formation and a ∼14.4‐fold improvement in desiccation tolerance (Figures S5 and S6).

### 2.4 Coupled Physical Buffering and Transcriptional Remodeling

To elucidate the comprehensive mechanism by which the AEM confers desiccation tolerance, we decoupled its physical protective role from its capacity to induce beneficial biological adaptation. We first investigated the physical reinforcement provided by the coating during the dehydration process.

**FIGURE 5:**
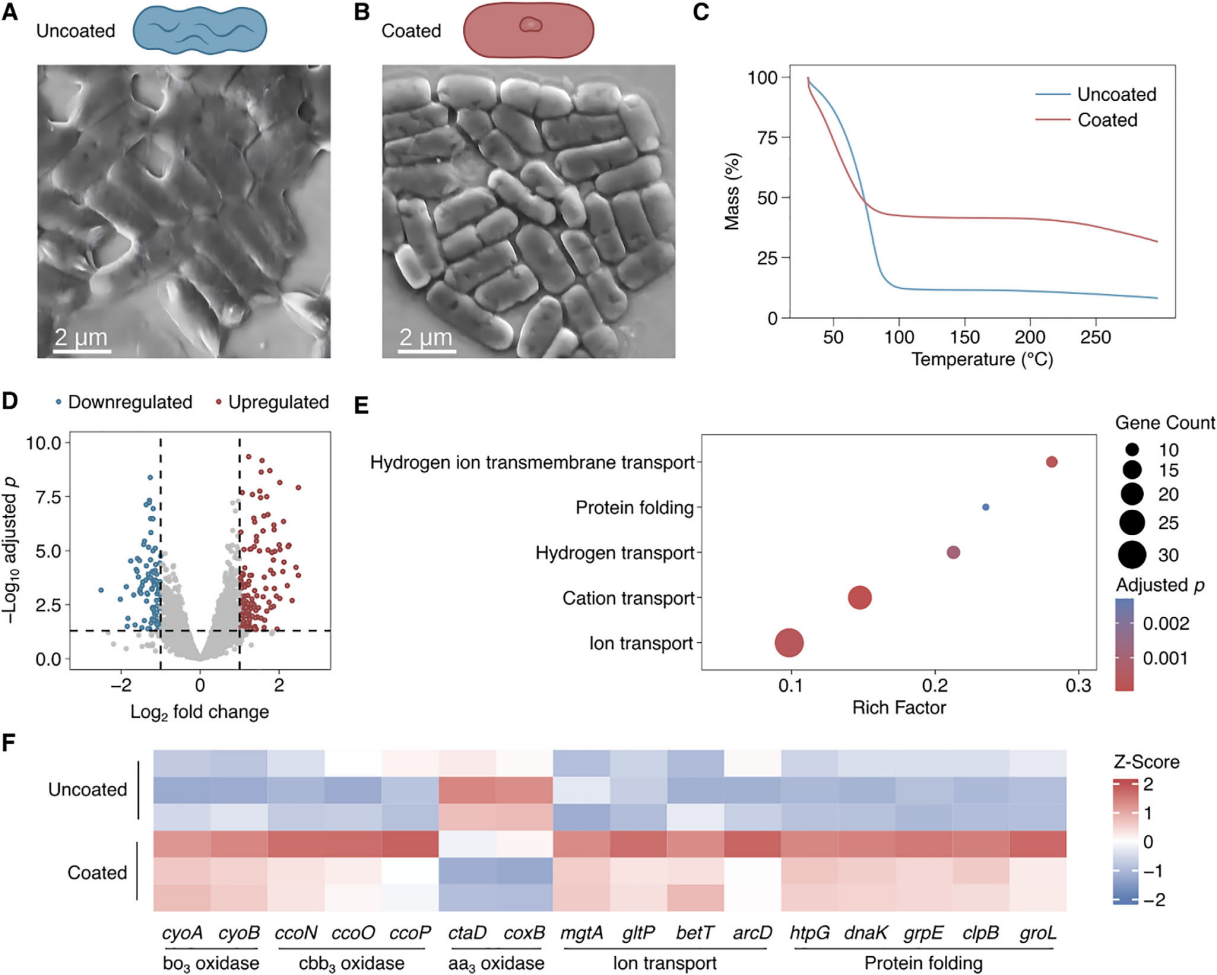
Physical protection and physiological adaptation mechanisms. (A, B) SEM images of bacteria after desiccation: (A) collapsed and aggregated uncoated cells vs. (B) intact, individually preserved AEM‐coated cells. (C) TGA curves showing slower dehydration during heating. (D) Volcano plot of RNA‐seq data showing differentially expressed genes (DEGs) in coated vs. uncoated bacteria. (E) GO enrichment analysis of DEGs. (F) Heatmap of representative DEGs, illustrating the shift in terminal oxidases and upregulation of ion transporters and proteostasis chaperones.

Morphological analysis using scanning electron microscopy (SEM) on unfixed cells revealed that the AEM provides critical structural integrity against the destructive capillary forces generated during drying (Figure 5A,B). While both populations exhibited reduced viability post‐desiccation, the coating fundamentally altered the mode of cellular death. Uncoated bacteria underwent catastrophic collapse, with cells fusing into amorphous aggregates. In stark contrast, AEM‐coated cells largely maintained their discrete, plump, rod‐shaped morphology with minimal visible membrane distortion. This structural preservation suggests the coating acts as a mechanical exoskeleton, mitigating the physical stress of water loss. The ability of the AEM to maintain this protective structure is underpinned by its superior hydration capacity. Thermogravimetric analysis (TGA) quantified this “hydration buffering” effect (Figure 5C and Figure S7). Upon heating, coated bacterial samples exhibited a slower rate of mass loss in the temperature range corresponding to water evaporation (30°C–100°C) and retained a higher residual mass. This confirms that the alginate‐rich matrix functions as an external water reservoir, moderating the efflux of water and preventing the rapid, catastrophic dehydration that typically ruptures the lipid bilayer.

Beyond physical shielding, RNA‐seq analysis revealed that the AEM triggers a profound and coordinated physiological remodeling in *P. fluorescens* (Figures S8 and S9). Of the 5656 genes detected, 103 were identified as significantly differentially expressed genes (DEGs), representing 1.3% of the total gene count (Figure 5D). This differential subset was characterized by a predominance of upregulated genes (*n* = 67) over downregulated ones (*n* = 36). Gene ontology (GO) enrichment analysis of genes upregulated in coated cells highlighted five dominant functional categories: hydrogen ion transmembrane transport, protein folding, hydrogen transport, cation transport, and general ion transport (Figure 5E). This transcriptional signature maps directly onto the principal failure modes of dehydration.

A heatmap of representative DEGs details this adaptive response (Figure 5F). First, we observed a metabolic shift in terminal oxidases: downregulation of aa3‐type oxidases (*ctaD*, *coxB*), associated with high‐oxygen planktonic growth, and concomitant upregulation of high‐affinity bo3‐ and cbb3‐type oxidases (*cyoA/B*, *ccoN/O/P*). This remodeling of the respiratory chain is a documented response to envelope stress and is geared toward maintaining proton‐motive force and energetic homeostasis under conditions of altered membrane hydration and ion flux [37]. Second, the strong induction of ion and osmolyte transporters (*gltP*, *arcD*, *betT*, and *mgtA*) addresses the ionic and osmotic imbalance caused by water loss [38]. These systems work synergistically to stabilize cytoplasmic pH, import compatible solutes like betaine, and adjust cation levels (e.g., Mg^2+^) to support membrane integrity. Finally, a robust activation of the core proteostasis network was evident, with significant upregulation of chaperones and disaggregases (*dnaK*, *grpE*, *groL*, *htpG*, and *clpB*). This preemptive bolstering of protein folding and repair capacity equips the cell to withstand the widespread proteotoxic stress that is a primary cause of lethality during desiccation. Collectively, the AEM demonstrates a superior capacity to prime bacteria for abiotic resilience by synergistically coupling extrinsic physical buffering with transcriptional remodeling.

### 2.5 Functional Translation: Interfacial Adhesion and Biocontrol Efficacy

The transformation of the bacterial cell surface, characterized by enhanced desiccation tolerance and adhesiveness, translated directly into superior performance in a complex, real‐world application: the colonization and protection of corn seeds. This scenario subjects a microbial inoculant to acute desiccation stress during both storage and seed germination, representing a stringent test of our material's resilience [39].

**FIGURE 6:**
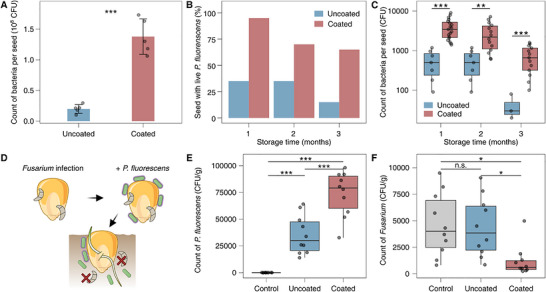
Enhanced seed adhesion and biocontrol efficacy. (A) Initial bacterial adhesion density to corn seeds (*n* = 5). Coated bacteria show ∼7‐fold higher attachment. An unpaired two‐tailed Student's t‐test was used to compare the means of the two groups. (B) Percentage of seeds retaining viable bacterial populations over 3 months of storage. (C) Absolute count of viable bacteria per seed during storage. An unpaired two‐tailed Student's t‐test was used to compare the means of the coated and uncoated groups at each time point. (D) Schematic of the biocontrol experiment against *Fusarium* infection. (E) Rhizosphere colonization density of *P. fluorescens* on corn seedlings (*n* = 10). Statistical analysis was performed using one‐way ANOVA followed by Tukey's multiple‐comparison test. (F) Fungal burden on seedlings (*n* = 10). Coated bacteria significantly suppress *Fusarium* growth. Statistical analysis was performed using one‐way ANOVA followed by Tukey's multiple‐comparison test. Data are presented as mean ± SD. *p < 0.05, **p < 0.01, ***p < 0.001.

We first quantified the adhesive advantage conferred by the AEM. The coated *P. fluorescens* exhibited robust attachment to corn seeds, achieving an initial adhesion density of approximately 1.4 × 10^8^ CFU per seed (Figure 6A). This represents a nearly 7‐fold increase over the uncoated strain, which aligns directly with the increased adhesion force measured by AFM (Figure 3H). Furthermore, the AEM provided exceptional long‐term stability to bacteria under desiccation on the seed surface. During a 3‐month shelf‐storage simulation, the coating acted as a preservative shield. Over 65% of seeds treated with coated bacteria retained viable populations, compared to only ∼15% of seeds treated with uncoated bacteria (Figure 6B). When quantified, the protective effect was even more striking: after 3 months, an average of 900 viable coated bacteria remained per seed, while only about 30 uncoated bacteria survived (Figure 6C). This confirms the dual functionality of the AEM: serving as both an adhesive for initial attachment and a resilient buffer for long‐term preservation.

The ultimate validation of this living material lies in its functional efficacy within a competitive biological environment. We evaluated its performance as a biocontrol agent against the seed‐borne fungal pathogen *Fusarium oxysporum* [40], which causes devastating wilt and rot in corn. Seeds were first infected with *F. oxysporum* and then treated with either uncoated or AEM‐coated *P. fluorescens* before being planted in soil (Figure 6D). After 7 days, the coated bacteria had successfully colonized the spermosphere and rhizosphere, achieving a robust population density of 8 × 10^4^ CFU/g of soil despite the initial desiccation stress (Figure 6E). This established population outcompeted the pathogen via competitive exclusion and resource dominance [40]. Consequently, seeds treated with coated bacteria showed significantly suppressed fungal growth, reducing the *Fusarium* burden to below 1000 CFU/g (Figure 6F). In contrast, seeds treated with uncoated bacteria failed to control the pathogen, which proliferated to levels (∼4000 CFU/g) comparable to the untreated infected control. Importantly, confrontation assays showed that coated and uncoated *P. fluorescens* displayed comparable antifungal activity when tested at equal cell numbers, while the coating materials alone showed no detectable inhibition against *Fusarium* (Figure S10), indicating that the improved biocontrol performance arises from enhanced bacterial retention and survival rather than altered intrinsic antifungal potency.

To further evaluate plant performance at the seedling stage, we measured the activities of antioxidant enzymes associated with pathogen‐induced oxidative stress, including catalase (CAT), superoxide dismutase (SOD), and peroxidase (POD). Because the severity of *Fusarium* infection does not always translate directly into macroscopic traits such as plant height or shoot fresh mass, particularly at early stages, these biochemical markers provide a more sensitive readout of plant physiological status. Seedlings grown from seeds treated with AEM‐coated *P. fluorescens* exhibited significantly lower CAT, SOD, and POD activities than the uncoated bacteria group, indicating reduced oxidative stress under *Fusarium* challenge (Figure S11). These results further support that the AEM‐coated bacteria alleviate pathogen‐associated stress and improve seedling resilience.

## 3 Conclusion

In summary, we have established a generalizable, biomimetic strategy to engineer an AEM that endows sensitive bacteria with exceptional abiotic resilience. Our design leverages the conformational transition of a globular protein into a β‐sheet‐rich, amyloid‐like state enabled by disulfide bond reduction to dramatically enhance its interfacial adhesive function. This restructured protein acts as a dynamic, cationic scaffold that electrostatically recruits an anionic polysaccharide (alginate) to the bacterial surface, driving the self‐assembly of a conformal, hydrated nanofilm directly at the cell envelope. The resulting AEM operates through a coherent, dual‐mechanism of protection. Physically, it forms a viscoelastic hydration buffer that retards water loss, dissipates mechanical stress, and prevents membrane rupture during desiccation. Biologically, it triggers a pre‐adaptive transcriptional program that remodels the cell's respiration, ion homeostasis, and proteostasis networks, proactively countering the primary causes of dehydration‐induced death. By ensuring the delivery, survival, and functionality of microbial payloads under practical, stressful conditions, as demonstrated by enhanced seed colonization and effective biocontrol against *Fusarium*, this strategy opens new avenues for designing robust living materials for applications ranging from sustainable agriculture to biomanufacturing and living therapeutics.

## 4 Experimental Section

### 4.1 Bacterial Culture

*Pseudomonas fluorescens* was used as the model plant‐beneficial bacterium. Cells were routinely cultured in LB medium at 30°C with shaking at 180 rpm until reaching the mid‐exponential growth phase. Bacterial cells were harvested by centrifugation (5,000 *g*, 5 min), washed twice with sterile phosphate‐buffered saline (PBS, pH 7.4), and resuspended to the desired cell density for subsequent surface modification experiments.

### 4.2 Preparation of Denatured Lysozyme and Alginate Solutions

To prepare the denatured lysozyme stock solution (5 mg/mL), 20 mg of hen egg‐white lysozyme was dissolved in 2 mL of sterile deionized water to yield a 10 mg/mL concentration. Concurrently, a 10 mm solution of reduced glutathione (GSH) was prepared by dissolving 6.2 mg of GSH in 2 mL of sterile deionized water. Equal volumes of the lysozyme and GSH solutions were combined and incubated at room temperature for 30 min to facilitate protein denaturation. Separately, sodium alginate solutions (4 mg/mL) were prepared by dissolving 4 mg of the polysaccharide powder in 1 mL of sterile deionized water.

### 4.3 Interfacial Assembly of the Artificial Extracellular Matrix (AEM) on Bacterial Surfaces

The AEM was constructed via the electrostatic co‐assembly of denatured lysozyme and sodium alginate directly onto the bacterial cell envelope. Briefly, a 0.5 mL suspension of *P. fluorescens* (OD_600_ = 4.0) was first mixed with 0.5 mL of sodium alginate (4 mg/mL) and vortexed for 20 s to ensure uniform distribution. Subsequently, 0.4 mL of the previously prepared denatured lysozyme (5 mg/mL) was added to the mixture and vortexed for an additional 20 s. The final volume of the reaction system was adjusted to 2 mL using phosphate‐buffered saline (PBS), resulting in a bacterial density of OD_600_ = 1.0, an alginate concentration of 1 mg/mL, and a denatured lysozyme concentration of 1 mg/mL. The assembly process was allowed to proceed at room temperature for 15 min under gentle agitation. Following incubation, the AEM‐modified bacteria were collected via two cycles of centrifugation (5000 rpm for 3 min) and washed thoroughly with PBS to remove any unbound biopolymers. For optimization, the mass ratios of lysozyme to alginate were systematically varied (1:0.1, 1:0.5, 1:1, and 1:2) to identify the stoichiometric balance required for maximal desiccation tolerance.

Control experiments were performed using uncoated bacteria subjected to identical washing and centrifugation steps but without the addition of polymers. To confirm the surface‐specific nature of the assembly, polymer mixtures were also incubated in the absence of bacteria; while bulk precipitates formed rapidly in these cell‐free solutions due to uncontrolled complexation, the presence of bacteria successfully templated the assembly at the interface, maintaining a homogeneous solution.

### 4.4 Characterization of Surface Modification by Fluorescence Microscopy and Flow Cytometry

To confirm the successful integration and spatial distribution of the AEM components on the bacterial surface, multi‐channel fluorescence imaging and quantitative flow cytometry were employed. The individual components of the AEM were selectively tracked using specific fluorescent probes. The presence of β‐sheet‐rich, amyloid‐like denatured lysozyme was visualized by staining the modified cells with Thioflavin S (ThS). A 100 µL volume of ThS solution (1 mg/mL) was added to the 2 mL reaction system and incubated for 10 min (or up to 30 min for quantitative assays) at room temperature in the dark. Sodium alginate was tracked by pre‐labeling the polymer with 5‐aminofluorescein. This was achieved through a 3 h carbodiimide‐mediated reaction involving EDC and NHS, followed by purification through a desalt column. Following staining, the cells were washed three times with sterile water or PBS to eliminate background interference from unbound dyes and imaged using an IX73 fluorescence microscope. Coated bacteria exhibited distinct blue fluorescence from ThS‐labeled protein and green fluorescence from FITC‐labeled alginate, whereas uncoated controls showed negligible signal. For high‐throughput quantification of the coating efficiency, stained bacterial populations were analyzed using a CytoFLEX flow cytometer.

### 4.5 Transmission Electron Microscopy (TEM)

The ultrastructure of the bacterial surface coating was examined by TEM. Uncoated and surface‐modified *P. fluorescens* cells were collected by centrifugation and gently washed with PBS. Samples were fixed with glutaraldehyde. After dehydration through a graded ethanol series, cells were deposited on a copper grid for imaging. TEM was performed to visualize the presence, continuity, and thickness of the artificial extracellular matrix at the bacterial envelope.

### 4.6 Atomic Force Microscopy (AFM) Measurements

AFM was employed to characterize the mechanical and adhesive properties of bacterial surfaces. Uncoated and coated bacterial cells were immobilized on freshly cleaved mica substrates. Force–distance curves were acquired under ambient conditions using a calibrated cantilever. The Young's modulus of individual bacterial cells was calculated from nanoindentation data using an appropriate contact mechanics model. Adhesion forces were extracted from the retraction curves. Multiple cells were analyzed for each condition to ensure statistical robustness.

### 4.7 Desiccation Tolerance Assays

To evaluate desiccation tolerance, uncoated and surface‐modified bacterial suspensions were deposited onto sterile substrates and subjected to controlled drying conditions. The bacterial suspension (OD_600_ of 1.0; 7.3 × 10^8^ CFU/mL) was loaded in 10 µL samples and dried at 35°C and 35% relative humidity for defined time intervals. After desiccation, bacteria were rehydrated with sterile PBS and gently resuspended. Viable cell counts were determined by serial dilution and plating on LB agar, followed by incubation at 30°C.

For optimization studies, desiccation survival was compared across different lysozyme‐to‐alginate mass ratios. Time‐dependent desiccation tolerance was assessed by extending drying durations up to 48 h. Survival rates were calculated by normalizing colony‐forming units (CFUs) after drying to those of non‐dried controls.

### 4.8 Scanning Electron Microscopy (SEM)

To visualize the structural consequences of dehydration at the cellular level, the morphological integrity of both uncoated and AEM‐modified *P. fluorescens* was evaluated using SEM. To ensure that the observed changes accurately represented desiccation‐induced damage rather than artifacts of chemical stabilization, the samples were prepared without the use of traditional glutaraldehyde fixation or ethanol dehydration. A 5 µL aliquot of the bacterial suspension was deposited directly onto a silicon wafer. The samples were then subjected to a controlled drying process at 35°C for 6 h in a constant‐temperature oven, allowing for in situ dehydration.

### 4.9 Thermogravimetric Analysis (TGA)

Thermogravimetric analysis was performed to quantify the dehydration behavior and water‐retention capacity of uncoated and surface‐modified bacterial samples. Following collection via centrifugation (6000 rpm, 3 min), the supernatant was discarded and excess extracellular water was carefully blotted with absorbent paper to ensure that only intracellular and matrix‐associated water was quantified during the analysis. The resulting bacterial pellets were immediately transferred to TGA crucibles and measured under a nitrogen atmosphere with a constant heating rate of 10°C/min, ranging from room temperature to 300°C. Mass loss profiles were recorded as a function of temperature, and the derivative thermogravimetric (DTG) curves were calculated to identify specific dehydration rates. Water loss in the low‐temperature region (30 C–100°C) was specifically analyzed to assess the hydration buffering effect provided by the artificial extracellular matrix.

### 4.10 RNA Extraction and Sequencing

To investigate transcriptional responses induced by surface modification, uncoated and AEM‐coated *P. fluorescens* cells were prepared under identical conditions. After coating, bacterial cells were subjected to controlled desiccation at 35°C and 35% RH for 4 h, and were then rapidly rehydrated within 1 min. Cells were immediately harvested by centrifugation, and total RNA was extracted using a commercial bacterial RNA extraction kit according to the manufacturer's protocol. Residual genomic DNA was removed by DNase treatment. RNA integrity and concentration were assessed by agarose gel electrophoresis and spectrophotometric analysis. Only high‐quality RNA samples were used for subsequent sequencing.

RNA sequencing libraries were constructed from purified total RNA following standard protocols for prokaryotic transcriptome analysis with the aid of Shanghai Biotechnology Corporation. Ribosomal RNA was removed prior to library construction to enrich for messenger RNA. Sequencing was performed on an Illumina platform to generate paired‐end reads. Raw sequencing data were subjected to quality control to remove adapter sequences, low‐quality reads, and contaminating sequences. The raw RNA‐seq data have been deposited in the NCBI Sequence Read Archive under BioProject PRJNA1456127.

Clean reads were mapped to the reference genome of *P. fluorescens* using a suitable alignment algorithm. Gene expression levels were quantified and normalized. Differentially expressed genes between AEM‐coated and uncoated bacteria were identified using a statistical framework based on count data. Genes with an adjusted *p*‐value below 0.05 and an absolute fold‐change exceeding 2 were considered significantly differentially expressed. A complete list of detected transcripts, including log2 fold change and adjusted *p*‐values, is provided in Data S1.

### 4.11 Seed Adhesion Assay

To quantify the adhesive capacity and interfacial stability of the AEM on biological substrates, a multi‐modal recovery protocol was developed using surface‐sterilized corn seeds as a model system. Corn seeds were disinfected by pre‐soaking in sterile water for 12 h, followed by immersion in 2% sodium hypochlorite for 20 min, triple washing with 75% ethanol for 5 min each, and rinsing with sterile water five times to remove residual chemicals. Uncoated and AEM‐coated *P. fluorescens* suspensions were incubated with the seeds under gentle agitation (120 rpm) for 2 h at room temperature. Following incubation, the seeds were subjected to a stringent triple‐washing procedure with 50 mL of sterile PBS to eliminate loosely associated or entrapped cells, ensuring that only firmly adhered bacteria remained. To recover these interface‐bound cells, each seed was immersed in a specialized recovery buffer consisting of PBS supplemented with 0.05% (v/v) Tween 80 and 10 mm EDTA. The non‐ionic surfactant Tween 80 was employed to reduce interfacial tension and disrupt hydrophobic interactions, while EDTA served as a chelating agent to sequester stabilizing divalent cations and soften the electrostatically assembled AEM. Complete detachment was then achieved through a combined regimen of low‐temperature ultrasonication (40 kHz, 5 min) and high‐speed vortexing for 60 s. The resulting suspensions were serially diluted and quantified via the spread‐plate method on LB agar, with the results expressed as CFU per seed.

### 4.12 Seed Storage Stability Assay

To evaluate the long‐term survival and shelf‐stability of the bacterial inoculants on a biological substrate, a storage stability assay was conducted using corn seeds over a 3‐month period. Corn seeds were coated with either uncoated or AEM‐modified *P. fluorescens* as described in the interfacial assembly protocol. Following the coating process, the seeds were air‐dried and stored under ambient conditions (20 C ± 2°C and a relative humidity of 45% ± 5%) to simulate a standard shelf‐storage environment. At predetermined time points throughout the 3‐month duration, seeds were randomly sampled and rehydrated in sterile PBS. The surviving bacteria were then detached from the seed surfaces and quantified using the standard serial dilution and spread‐plating method on LB agar. The stability of the inoculants was assessed by recording both the percentage of seeds retaining viable bacterial populations and the absolute count of viable CFU per seed.

### 4.13 Confrontation Assay Against Fusarium

To assess antifungal activity, a plate confrontation assay was performed on PDA plates. A mycelial plug of *F. oxysporum* was inoculated at the center of each plate, and coated or uncoated *P. fluorescens* suspensions were spotted symmetrically at equal distances from the fungal plug. Before inoculation, the bacterial suspensions were normalized to the same cell density to ensure a fair comparison of intrinsic antifungal activity. Denatured lysozyme and sodium alginate without bacteria were included as a material‐only control. After incubation at 28°C for several days, fungal growth inhibition was evaluated by the presence and size of the inhibition zone.

### 4.14 Biocontrol Assay Against Fusarium

To evaluate the functional performance of the AEM in a practical agricultural setting, a biocontrol assay was conducted using corn seeds challenged by the fungal pathogen *Fusarium oxysporum*. Corn seeds were first surface‐disinfected to eliminate indigenous microflora. To simulate seed‐borne infection, the disinfected seeds were inoculated with *F. oxysporum* by immersion in a fungal spore suspension (1.0 × 10^6^ spores mL^−1^) for 2 h under gentle agitation. The infected seeds were then air‐dried in a sterile laminar flow hood. The pathogen‐infected seeds were then treated with either uncoated *P. fluorescens* or AEM‐coated *P. fluorescens* (prepared at the optimized 1:1 mass ratio of denatured lysozyme to sodium alginate) by immersion in the respective bacterial suspensions (∼10^9^ CFU mL^−1^) for 2 h. Untreated infected seeds served as a control group. All seeds were planted in sterilized soil. After 7 days of cultivation, the seedlings were harvested to assess microbial colonization and fungal burden. Soil adhering to the seed and roots was collected, weighed, and suspended in sterile PBS. The population densities of *P. fluorescens* and the residual *F. oxysporum* burden were quantified by serial dilution and plating on selective media (LB for *P. fluorescens* and PDA for *F. oxysporum*), with results expressed as CFU per gram of soil.

### 4.15 Antioxidant Enzyme Activity Assay in Corn Seedlings

Following the biocontrol experiment, the physiological response of corn seedlings to *Fusarium* infection was assessed by measuring antioxidant enzyme activities. After 7 days of growth, seedling tissues were harvested and homogenized in ice‐cold extraction buffer. After centrifugation, the resulting supernatants were collected for the analysis of catalase (CAT), superoxide dismutase (SOD), and peroxidase (POD) activities using standard commercial kits in accordance with the manufacturer's protocols. The enzyme activities were normalized to fresh tissue weight for comparison between groups.

### 4.16 Molecular Dynamics Simulation

The crystal structure of lysozyme (PDB ID: 5B1F) was obtained from the RCSB Protein Data Bank. Alginate pentamers and the membrane model, including lipopolysaccharide (LPS), were constructed using CHARMM‐GUI. The cell surface was composed of LPS, DOPE, and DOPG at a molar ratio of 1:3:1. The CHARMM36 force field was used to generate the topology files for all components. Each simulation system contained one lysozyme molecule, 13 alginate chains, and one lipid membrane in a simulation box of 5.5 × 5.5 × 28 nm^3^. The system was solvated with SPC water molecules and supplemented with 0.15 m NaCl to achieve charge neutrality and mimic physiological ionic strength. To represent the native and reduced/denatured forms of lysozyme, the presence or absence of disulfide bonds was specified during topology generation in GROMACS.

All systems were first subjected to energy minimization using the steepest descent algorithm for 50 000 steps. This was followed by a 100 ps equilibration in the canonical ensemble (NVT) at 310 K with positional restraints applied to the lysozyme, alginate chains, and membrane. The equations of motion were integrated using the leapfrog algorithm with a time step of 2 fs. Subsequent equilibration and production runs were carried out in the isothermal–isobaric ensemble (NPT) at 310 K and 1 bar, using the velocity‐rescale thermostat and the Parrinello–Rahman barostat. Electrostatic interactions were calculated using the particle mesh Ewald (PME) method with a cutoff of 1.0 nm, and van der Waals interactions were treated using a cutoff scheme with the same cutoff distance. After equilibration, unrestrained production simulations were performed for 200 ns under periodic boundary conditions in all three dimensions. All simulations were carried out using GROMACS 2022.2, and the trajectories were visualized using VMD.

### 4.17 Statistical Analysis

All experiments were performed with at least three independent replicates unless otherwise stated, and the exact sample size (*n*) for each analysis is provided in the corresponding figure legends. No data transformation or outlier exclusion was performed during analysis. Quantitative data are presented as mean ± standard deviation (SD). Statistical analyses were performed using R version 4.5.3. For comparisons between two groups, statistical significance was evaluated using an unpaired two‐tailed Student's t‐test. For comparisons among multiple groups, statistical significance was evaluated using one‐way analysis of variance (ANOVA), followed by Dunnett's post hoc test when each group was compared with a single reference group, or Tukey's multiple‐comparison test when all pairwise group comparisons were assessed. A p value < 0.05 was considered statistically significant. The statistical test used for each experiment is indicated in the corresponding figure legend.

## Conflicts of Interest

The authors declare no conflicts of interest.

## Supporting information




**Supporting file**: advs75937‐sup‐0001‐SuppMat.docx.

## Data Availability

The data that support the findings of this study are available on request from the corresponding author. The data are not publicly available due to privacy or ethical restrictions.
