# Supplementary material for: Molecular Engineering Mediated Interfacial Assembly as an Artificial Extracellular Matrix Remolds Bacteria With Enhanced Abiotic Resilience
Source: Adv Sci (Weinh). 2026 Jun 3:e75937. Online ahead of print. doi: 10.1002/advs.75937 (PMC13336912; doi:10.1002/advs.75937)
Supplement: Supplementary file 1 — Supporting file: advs75937‐sup‐0001‐SuppMat.docx. [file ADVS-9999-e75937-s001.docx]

**Supporting Information**

Molecular Engineering Mediated Interfacial Assembly as an Artificial Extracellular Matrix Remolds Bacteria with Enhanced Abiotic Resilience

Yuanyuan Wang,^†^ Zili Jia,^†^ Yang Li, Qitao Wang, Shuai Hou,* and Lei Liu*

Y. Wang, Z. Jia, Y. Li, Q. Wang, Prof. Dr. S. Hou, Prof. Dr. L. Liu
Institute for Advanced Materials, School of Materials Science and Engineering
Jiangsu University
Xuefu Road 301, Zhenjiang, China
E-mail: hou@ujs.edu.cn (S.H.); liul@ujs.edu.cn (L.L.)

[^†^] These authors contributed equally to this work.

**Table S1** List of chemical reagents

| **Reagent** | **Specification/Grade** | **Catalogue No.** | **Supplier** |
| --- | --- | --- | --- |
| Sodium alginate | CP Grade, 20 mPa·s (10 g/L, 20 °C) | 30164426 | Sinopharm Chemical Reagent |
| Lysozyme | Biotech Grade (chicken egg white) | L6051 | Macklin |
| Glutathione (GSH) | Biotech Grade | R917465 | Macklin |
| Thioflavin S (ThS) | Practical Grade | S19293 | Yuanye Bio |
| 5-Aminofluorescein | 95% Purity | A800825 | Macklin |


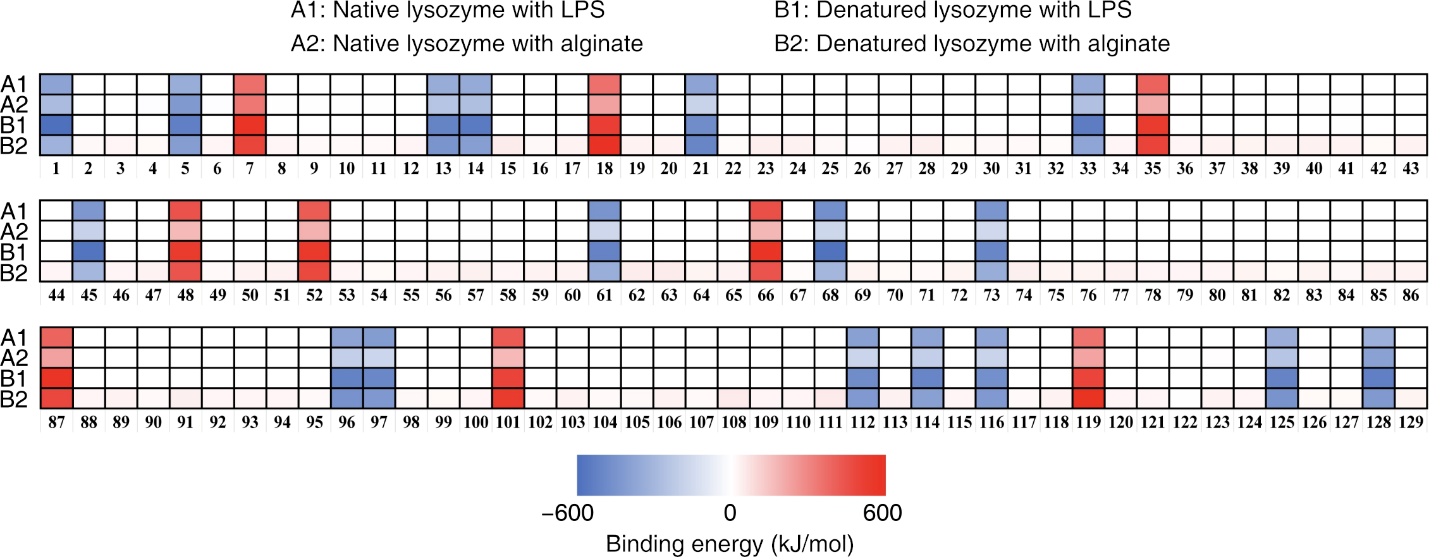


**Fig. S1** Per-residue binding energy analysis of lysozyme interaction with bacterial surface (LPS) and alginate. Heatmap illustrating the calculated binding energies (kJ/mol) for each individual amino acid residue of lysozyme across four different interaction scenarios: A1 (Native lysozyme with bacterial LPS), A2 (Native lysozyme with alginate), B1 (Denatured lysozyme with bacterial LPS), and B2 (Denatured lysozyme with alginate).


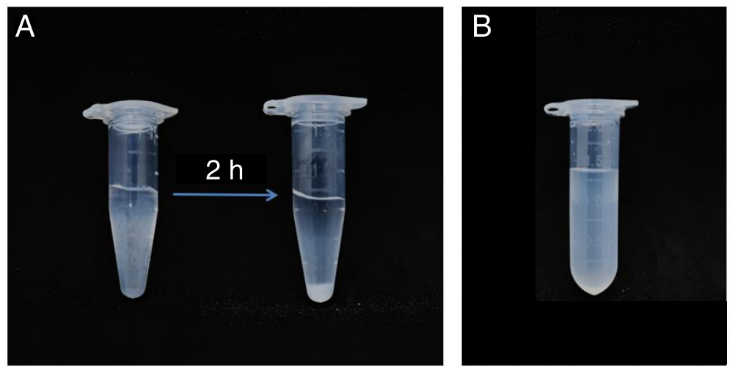


**Fig. S2** Macroscopic validation of bacterial surface-templated AEM assembly. (A) Uncontrolled bulk precipitation: Digital photographs of a solution containing denatured lysozyme and sodium alginate in the absence of bacteria. The formation of visible white precipitates after 2 hours indicates rapid, uncontrolled bulk electrostatic complexation between the oppositely charged biopolymers. (B) Surface-templated homogeneous assembly: Digital photograph of the same biopolymer mixture in the presence of *P. fluorescens*. The solution remains homogeneous and free of bulk precipitates, demonstrating that the bacterial cell envelope acts as a preferential nucleation site.


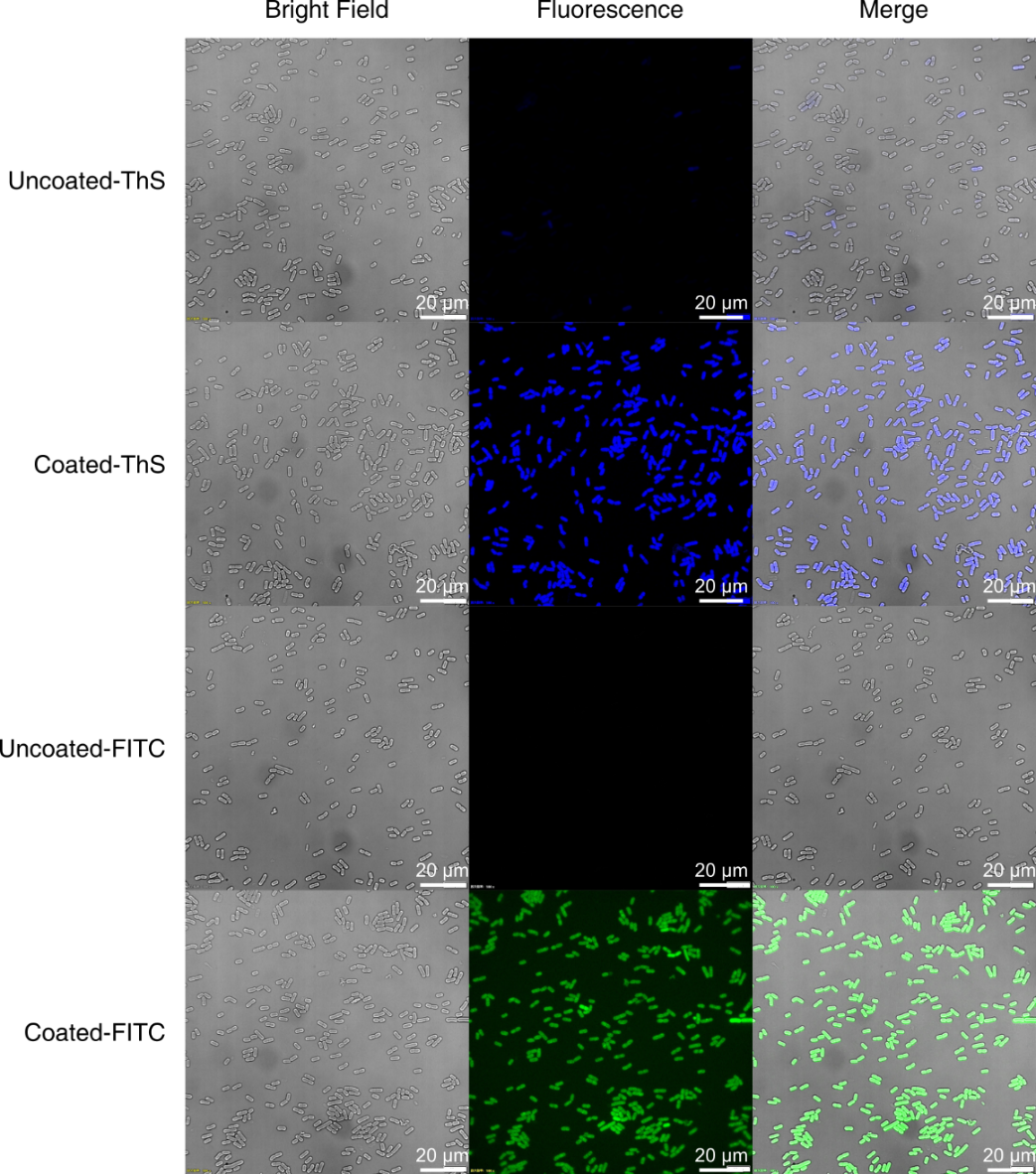


**Fig. S3** Fluorescence microscopic validation of AEM component assembly: Bright-field, fluorescence, and merged images of *P. fluorescens* cells comparing uncoated and AEM-coated bacteria stained for specific matrix components.

**
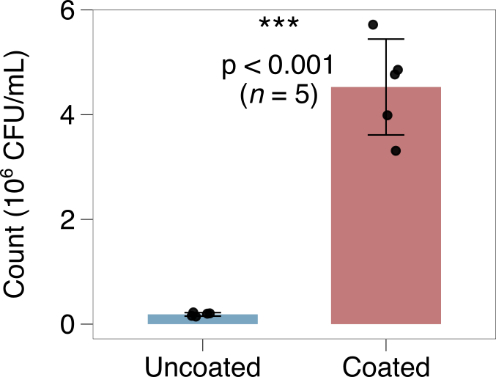
**

**Fig. S4** Survival rate of coated and uncoated *P. fluorescens* after desiccation at 40 °C and 35% RH for 4 h. Data are presented as mean ± SD (*n* = 5). An unpaired two-tailed Student's t-test was used to compare the means of two groups. **p* < 0.05, ***p* < 0.01, ****p* < 0.001.


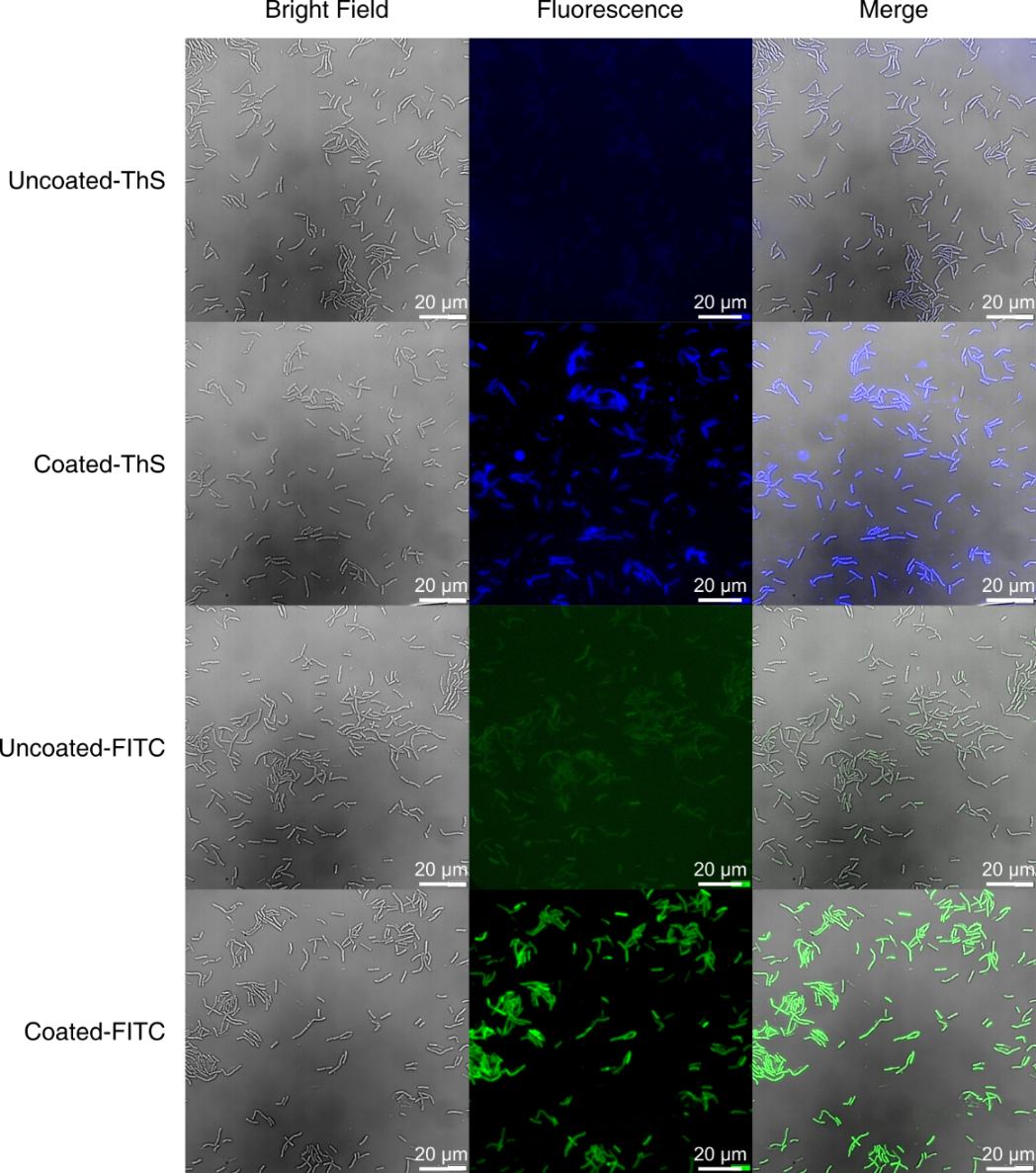


**Fig. S5** Fluorescence microscopic validation of AEM component assembly on *B. subtilis*: Bright-field, fluorescence, and merged images of *B. subtilis* cells comparing uncoated and AEM-coated bacteria stained for specific matrix components.


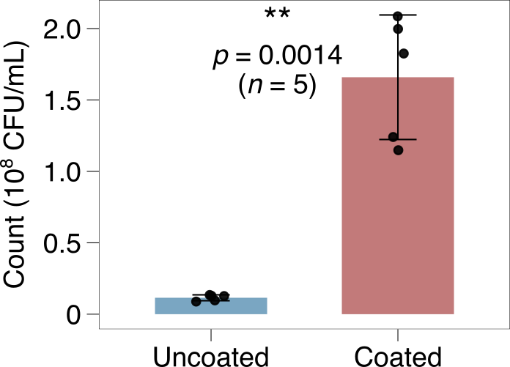


**Fig. S6** Survival rate of coated and uncoated *B. subtilis* after desiccation at 35 °C and 35% RH for 4 h. Data are presented as mean ± SD (*n* = 5). An unpaired two-tailed Student's t-test was used to compare the means of two groups. **p* < 0.05, ***p* < 0.01, ****p* < 0.001.


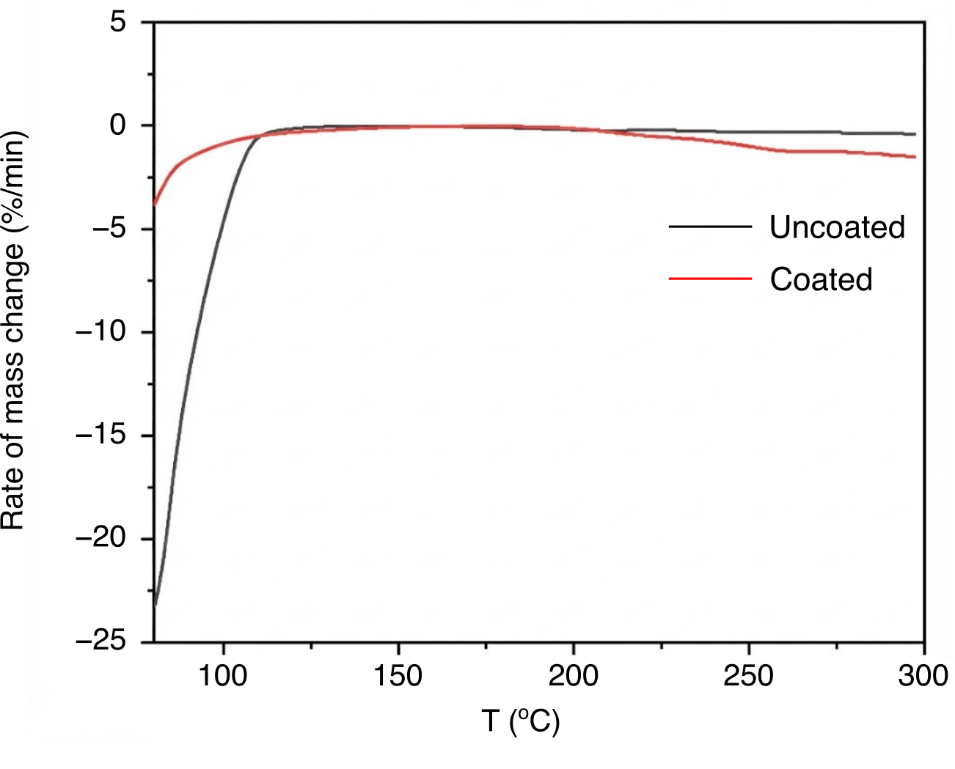


**Fig. S7** Rate of mass change relative to temperature for uncoated (black line) and AEM-coated (red line) bacteria.


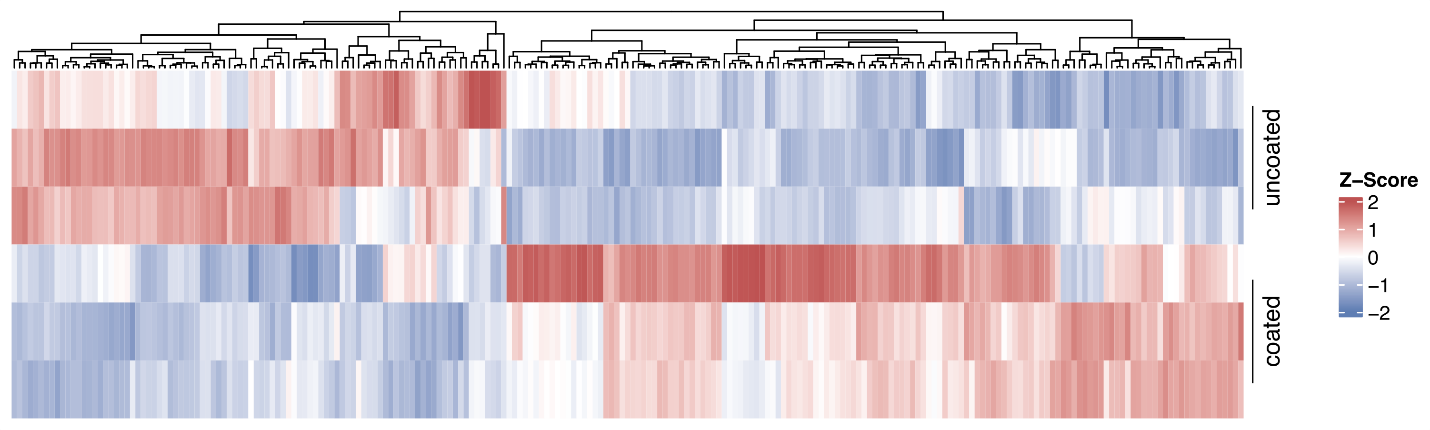


**Fig. S8** Hierarchical clustering and heatmap of differentially expressed genes comparing uncoated and AEM-coated *Pseudomonas fluorescens*.


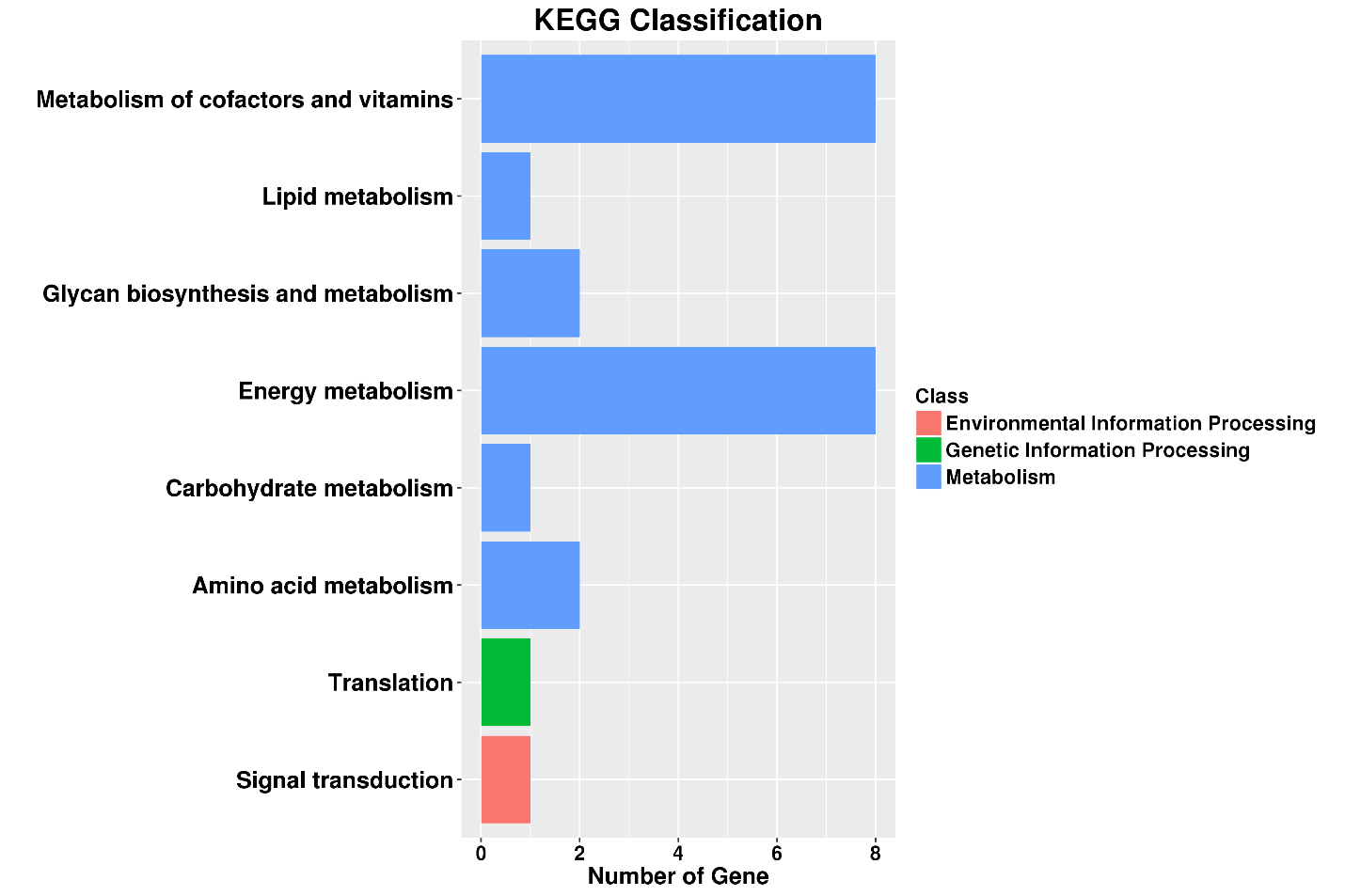


**Fig. S9** KEGG functional classification of differentially expressed genes.


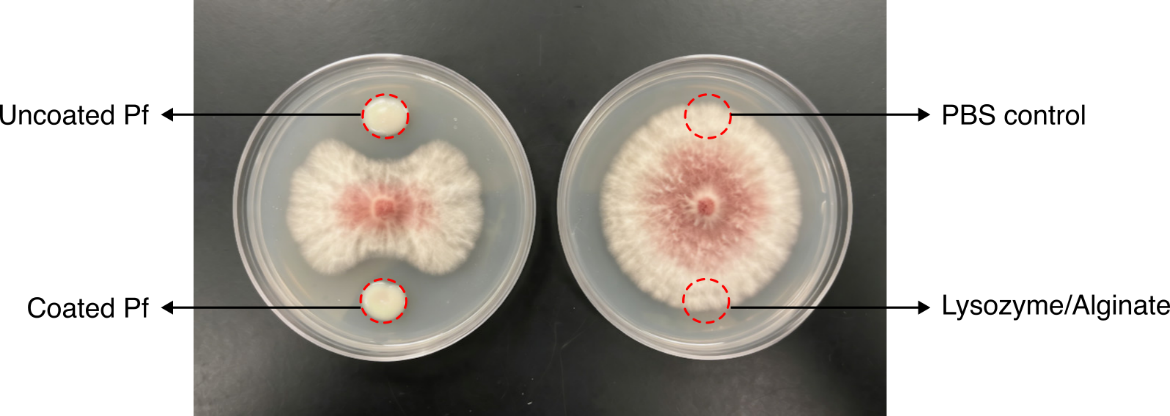


**Fig. S10** Confrontation assay against *F. oxysporum*.


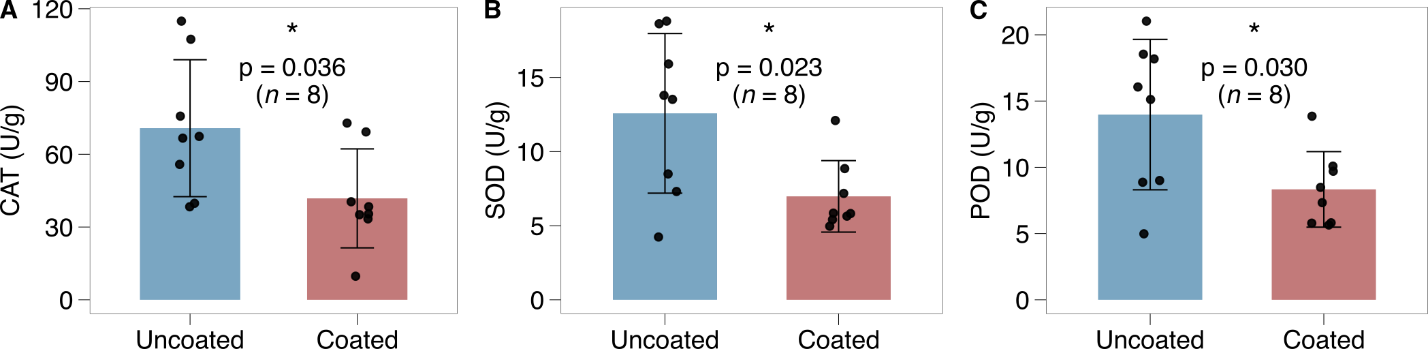


**Fig. S11** Antioxidant enzyme activity assay: (A) CAT activity; (B) SOD activity; and (C) POD activity. Data are presented as mean ± SD (*n* = 8). An unpaired two-tailed Student's t-test was used to compare the means of two groups. **p* < 0.05, ***p* < 0.01, ****p* < 0.001.
